# Launching *Advanced Biotechnology* to elevate biotechnology research across disciplines, from biomedicine to agriculture

**DOI:** 10.1007/s44307-023-00001-9

**Published:** 2023-10-26

**Authors:** Shi Xiao, Jianhua Yang, Jianguo He, Lianghu Qu, Songlin Chen

**Affiliations:** 1 State Key Laboratory of Biocontrol, Guangdong Provincial Key Laboratory of Plant Resources, School of Life Sciences, Sun Yat-Sen University, Guangzhou, 510275 China; 2 Yellow Sea Fisheries Research Institute, Chinese Academy of Fishery Sciences (CAFS), Key Laboratory for Sustainable Development of Marine Fisheries, Ministry of Agriculture, Qingdao, 266071 China

Here, we announce a new, open access (OA) journal, *Advanced Biotechnology*, which aims to promote research in biomedical and agricultural biotechnology. Biotechnology has a rich and fascinating history that dates back to ancient civilizations, who used methods such as fermentation and selective breeding to increase food production and improve agricultural outcomes. In the mid-20th century, major advances in genetics and molecular biology laid the foundation for modern biotechnology. Indeed, the development of recombination DNA technology in the 1970s marked a pivotal moment (Ishino and Ishino 2014), enabling humans to modify genes as they wished. This critical breakthrough led to the creation of genetically modified organisms and the emergence of biopharmaceuticals such as insulin produced using recombinant DNA technologies, which had a major impact on the treatment of diabetes (Kurtzhals et al. 2021). Likewise, the development of affinity chromatography in 1968 greatly facilitated the isolation and purification of numerous specific molecules (Rodriguez et al. 2020).

In recent decades, biotechnology has evolved rapidly, with breakthroughs in next-generation and single-molecule/single-cell sequencing, gene editing tools like clustered regularly interspaced short palindromic repeat (CRISPR)-Cas9, synthetic biology, artificial intelligence, and mRNA vaccines (Adli 2018; Ishino et al. 2018). These advances have given a tremendous boost to multiple fields, particularly agriculture and medicine.

The impact of biotechnology on medicine and agriculture has been truly transformative, offering novel approaches to enhance human health and agricultural productivity while simultaneously addressing the growing demands of a rapidly expanding global population. Today, biotechnology plays an integral role in tackling global challenges, offering innovative solutions for the fields of medicine and agriculture. In medicine, biotechnology has revolutionized the development of targeted drug therapies and advanced diagnostics, enabling the treatment of previously incurable genetic disorders and improving patient outcomes. Notably, the production of recombinant proteins and monoclonal antibodies has been instrumental in the creation of various vaccines, showcasing the potential of biotechnology to address emerging health crises. In agriculture, biotechnology has spurred the creation of plant varieties with enhanced resistance to pests, diseases, and environmental stressors, leading to markedly increased crop yields and food security. Additionally, biofortification and genetic engineering have allowed for the development of nutritionally enriched crops to help alleviate malnutrition and foster sustainable agricultural practices. Plant and animal biotechnology have made remarkable advances, but these advances tend to be published in niche, non-OA journals, limiting opportunities for cross-disciplinary innovation and collaboration.

Publishing research data is crucial for advancing our comprehension of modern biotechnology and its practical applications. The timely sharing of knowledge and experience from different fields within biotechnology will enhance research. While there are already commendable academic journals dedicated to biotechnology research, the expanding significance of this field, the opportunity for cross-disciplinary innovation, and the increasing volume of biotechnology papers call for the introduction of a fresh, innovative journal that covers biomedicine and agriculture. In light of these circumstances, we have launched a new international journal, *Advanced Biotechnology*, published in collaboration with Springer Nature and Sun Yat-sen University. This journal aims to fulfil the growing demand and address current gaps in the field of biotechnology.

*Advanced Biotechnology* publishes a wide range of content in the agricultural and biomedical sciences, including reviews, original research articles, letters, and commentaries, focusing on biotechnology-related studies that introduce novel methods and significant advancements. We aim to establish *Advanced Biotechnology* as a reputable peer-reviewed, fully OA journal. Authors will benefit from the journal’s affiliation with Springer Nature, which offers a streamlined initial submission process, a prompt and equitable peer-review procedure, expedited online publication, and impeccable production quality. Every manuscript undergoes a rigorous evaluation to ensure its quality and suitability for publication. Once accepted, papers are promptly published online after the completion of the page-proof stage, with the online publishing date serving as the official publication date.

Embracing an OA model, all published papers in *Advanced Biotechnology* will be freely accessible to scientists worldwide through the Springer Nature platform, ensuring wide dissemination of biotechnological discoveries and promoting the practical application of reported scientific findings. Publishing with a renowned publisher will enhance the visibility and reach of your research. Moreover, the journal will cover article processing charges for all published papers between 2023 and 2026, further facilitating authors’ contributions to the scientific community.

The Editors-in-Chief of *Advanced Biotechnology* are Prof. Song-Lin Chen, Prof. Jian-Guo He, and Prof. Liang-Hu Qu from Sun Yat-sen University. Their extensive expertise and distinguished contributions in the field of biotechnology ensure the journal’s high standards and commitment to excellence. Moreover, *Advanced Biotechnology* boasts a distinguished Editorial Board consisting of eminent international scholars who will assist in publishing your exceptional research. Members of the editorial board are also privileged to propose and organize timely special issues within their respective subfields, ensuring comprehensive coverage of cutting-edge topics and emerging trends in biotechnology.

Ultimately, through collaboration with the biotechnology research community, *Advanced Biotechnology* aims to foster academic exchange, serve as a primary source for new biotechnology breakthroughs, and inspire innovation and interdisciplinary interactions within the research community. We look forward to receiving submissions of your cutting-edge, high-quality research.
